# A Rare Case of Gastric Neuroendocrine Tumor in a Patient With Vitamin B12 Deficiency

**DOI:** 10.7759/cureus.68968

**Published:** 2024-09-09

**Authors:** Sava Nanda Gopal, Deepthi Vakati, Saranya Palanisamy, Kanimozhi David, Kannan Rajendran

**Affiliations:** 1 Internal Medicine, Saveetha Medical College and Hospital, Saveetha Institute of Medical and Technical Sciences, Saveetha University, Chennai, IND

**Keywords:** gastric neuroendocrine tumor, hepatitis b, incidental finding, macrocytic anemia, vitamin b12 deficiency

## Abstract

Vitamin B12 deficiency can result from gastric neuroendocrine tumors (GNETs), which are uncommon neoplasms frequently linked to hypergastrinemia and chronic atrophic gastritis. Here, we report the case of a 48-year-old vegetarian male from South India who presented with jaundice, fatigue, and gastrointestinal discomfort. He was diagnosed with macrocytic anemia, mild hepatomegaly, and significant vitamin B12 deficiency. An incidental upper gastrointestinal endoscopy revealed multiple gastric nodules, later confirmed as a well-differentiated GNET. The patient also had a high hepatitis B viral load. He was treated with vitamin B12 supplementation and underwent resection of the tumor followed by antiviral therapy for hepatitis B. Postoperative recovery was uneventful with improvements in anemia and liver function. This case emphasizes the importance of a multidisciplinary approach and thorough evaluation when addressing patients with vitamin B12 insufficiency, hepatitis B, and GNET.

## Introduction

A rare subclass of neoplasms emerging from enterochromaffin-like (ECL) cells found in the stomach lining are known as gastric neuroendocrine tumors (GNETs). Fewer than 1% of stomach cancers are GNETs, and they are often linked to hypergastrinemia, a disease marked by high gastrin levels that promote the growth of ECL cells and ultimately lead to tumor development [1]. Thought to be multifactorial in nature, Zollinger-Ellison syndrome, autoimmune gastritis, and atrophic gastritis are known to play a role in the development of GNETs [2].

As autoimmune gastritis is a known precursor to GNETs, vitamin B12 insufficiency is a clinically relevant disease frequently associated with it [3]. The breakdown of parietal cells in the stomach, which generate intrinsic factor, a glycoprotein essential for absorbing vitamin B12 in the terminal ileum, occurs as a result of autoimmune gastritis [4]. Red blood cells that are larger than normal in pernicious anemia, a kind of megaloblastic anemia, are caused by impaired vitamin B12 absorption due to a lack of intrinsic factors [5].

The connection between GNETs and vitamin B12 deficiency lies in their shared association with chronic atrophic gastritis [6]. This condition reduces gastric acid secretion, resulting in achlorhydria (absence of hydrochloric acid in the stomach). The compensatory hypersecretion of gastrin due to the lack of acidity drives ECL cell hyperplasia, which can eventually lead to the formation of neuroendocrine tumors in the gastric mucosa [7].

Although hypergastrinemia and GNETs have been linked, only a few cases of vitamin B12 deficiency, especially in hospitalized patients, have been reported to result in GNET development. This unusuality highlights the need for improved clinical knowledge and a deeper comprehension of the pathophysiology connection between these disorders [8].

This case study describes a patient who developed a vitamin B12 deficiency and a stomach neuroendocrine tumor by coincidence. This case attempts to clarify the clinical presentation, diagnostic challenges, and possible treatment approaches for this complicated comorbidity via an in-depth analysis.

## Case presentation

A 48-year-old male from South India, who followed a strict vegetarian diet, presented with symptoms that began with gradual-onset fatigue and weakness, yellowish discoloration of the eyes, gastrointestinal discomfort, and difficulty in passing stools, which worsened over one month. Initial blood tests revealed severe vitamin B12 deficiency and positive hepatitis B surface antigen (HBsAg), further complicating the clinical picture.

On general examination, the patient appeared clinically stable with vital signs within the normal limits. However, pallor was noted, along with mild icterus. As diagnostic testing progressed, an abdominal CT scan showed a thickening in the greater curvature of the stomach. An upper gastrointestinal endoscopy revealed multiple sessile nodular lesions in the gastric fundus, later confirmed as GNET via a biopsy.

Laboratory findings

Laboratory investigations showed macrocytic anemia, with a mean corpuscular volume (MCV) of 118.8 fL (normal range: 60-100 fL). The hemoglobin level was significantly reduced at 6.8 g/dL (normal range for men: 13.8-17.2 g/dL). Liver function tests indicated mild elevations in aspartate aminotransferase at 78 U/L (normal range: 10-40 U/L) and alanine aminotransferase at 43 U/L (normal range: 7-56 U/L). Serum vitamin B12 was markedly low at less than 159 pg/mL (normal range: 200-900 pg/mL). Notably, the patient tested positive for HBsAg, with a viral load of 22,726 copies/mL (normal viral load: <20,000 copies/mL) (Table 1).

**Table 1:** Laboratory findings and diagnostic results of the patient with neuroendocrine tumor and vitamin B12 deficiency.

Test	Patient’s result	Normal range	Interpretation
Hemoglobin	6.8 g/dL	13.8–17.2 g/dL (men)	Low: Suggestive of anemia
Mean corpuscular volume	118.8 fL	80–100 fL	High: Macrocytic anemia
White blood cell count	Normal	4,000–11,000 cells/µL	Normal
Platelet count	Normal	150,000–450,000 cells/µL	Normal
Liver function tests			
Aspartate aminotransferase	78 U/L	10–40 U/L	Elevated
Alanine aminotransferase	43 U/L	7–56 U/L	Mildly elevated
Serum vitamin B12	<159 pg/mL	200–900 pg/mL	Low: Indicative of vitamin B12 deficiency
Hepatitis B surface antigen	Positive	Negative	Positive
Hepatitis B viral load	22,726 copies/mL	<20,000 copies/mL (low)	High: Active viral replication
Creatinine	0.6 mg/dL	0.7–1.3 mg/dL (men)	Normal
Urea	12 mg/dL	7–20 mg/dL	Normal
Peripheral smear	Dimorphic anemia	Normal red blood cell morphology	Abnormal: Mixed anemia
Stool occult blood test	Negative	Negative	Normal

Imaging and diagnostic studies

**Figure 1:**
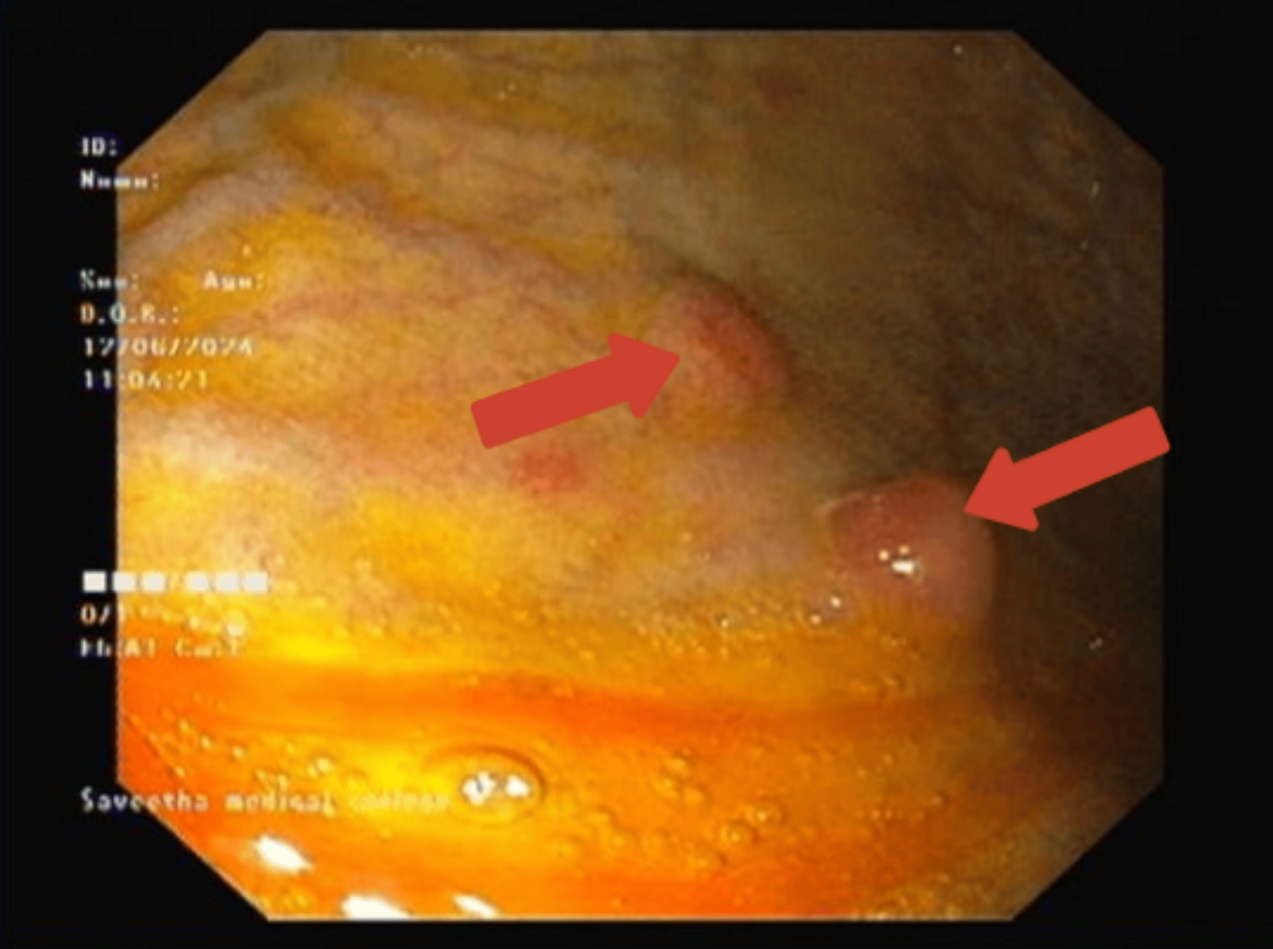
Upper gastrointestinal endoscopy showing multiple sessile nodular lesions. Upper gastrointestinal endoscopy showing multiple small, round, reddish nodules with a smooth surface in the gastric antrum. The nodules are indicative of possible neuroendocrine tumor involvement with the surrounding mucosa appearing normal.

Given the laboratory findings, an upper gastrointestinal endoscopy was performed, revealing multiple sessile nodular lesions in the gastric fundus, which are characteristic of GNETs (Figure 1). These lesions were smooth and erythematous and stood out due to their distinct appearance against the normal surrounding mucosa. The endoscopic findings were pivotal in guiding the decision to proceed with a biopsy for a definitive diagnosis. A biopsy of these lesions indicated the presence of a well-differentiated neuroendocrine tumor. Further imaging with a contrast-enhanced CT (CECT) scan confirmed thickening in the greater curvature of the stomach, while a PET-DOTATATE gallium scan showed an isolated nodule in the same region.

**Figure 2:**
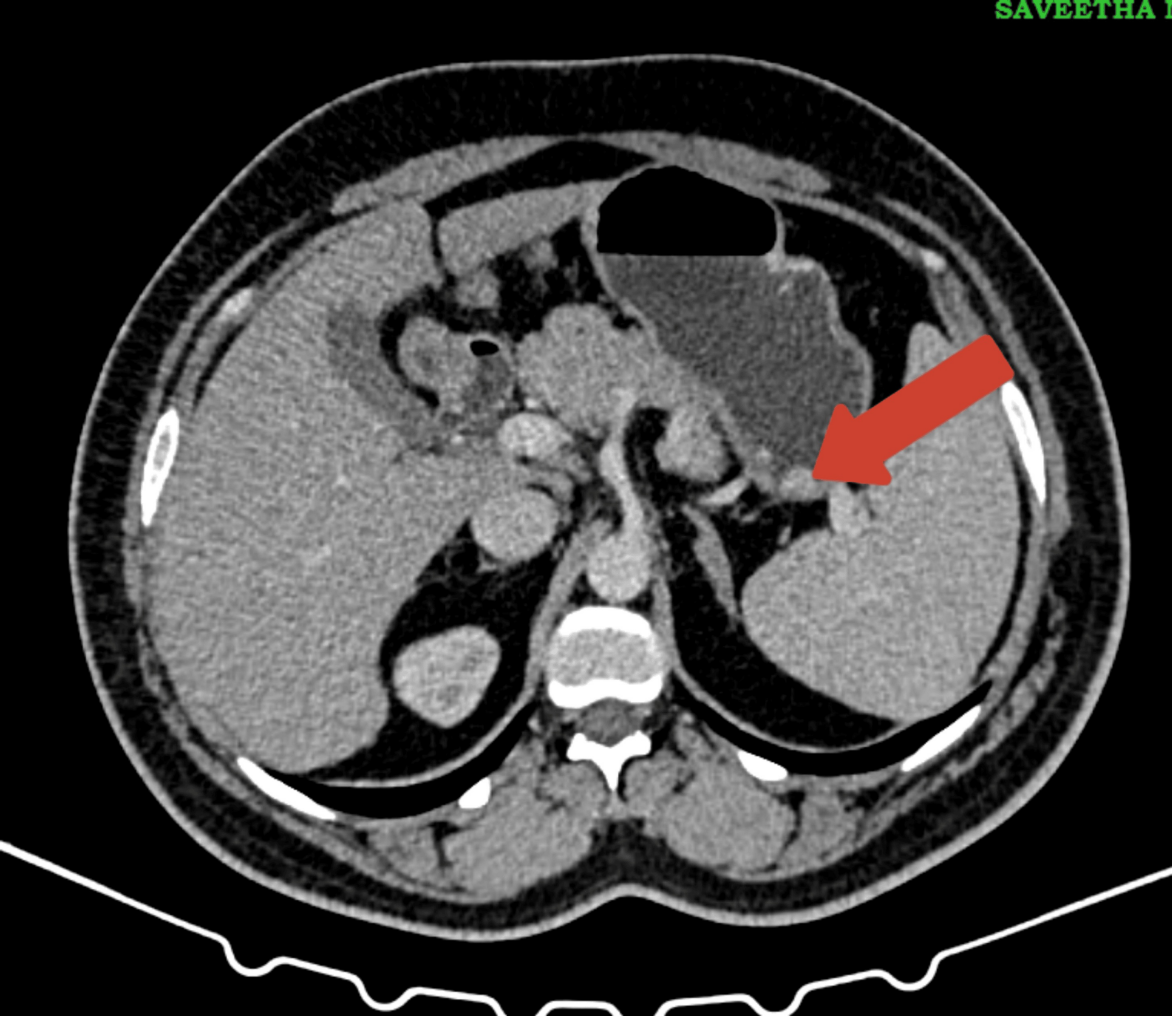
Contrast-enhanced CT (CECT) of the abdomen showing thickening in the greater curvature of the stomach. The axial CECT image of the abdomen highlights a thickened segment of the greater curvature of the stomach (indicated by the red arrow).

A CECT scan further highlighted a thickening in the greater curvature of the stomach, which raised the suspicion of tumor invasion, prompting more detailed imaging and biopsy (Figure 2).

Histopathological findings

**Figure 3:**
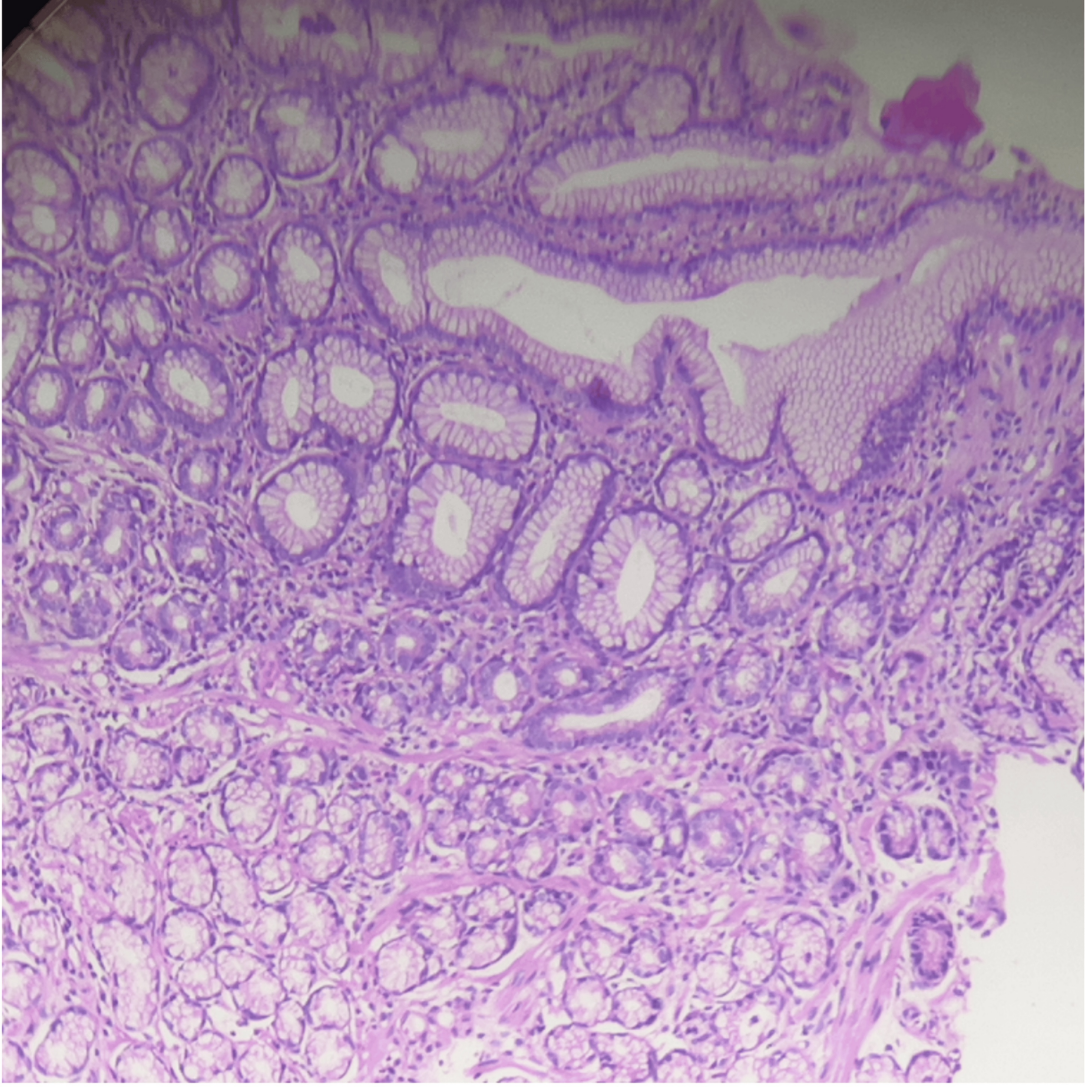
Histopathological section of glandular tissue showing glandular architecture and cellular morphology (hematoxylin and eosin (H&E) stain, high magnification). Histopathological section of glandular tissue stained with H&E, demonstrating the typical architecture of glandular structures. The image shows well-formed glands lined by columnar epithelial cells with basal nuclei and clear cytoplasm.

**Figure 4:**
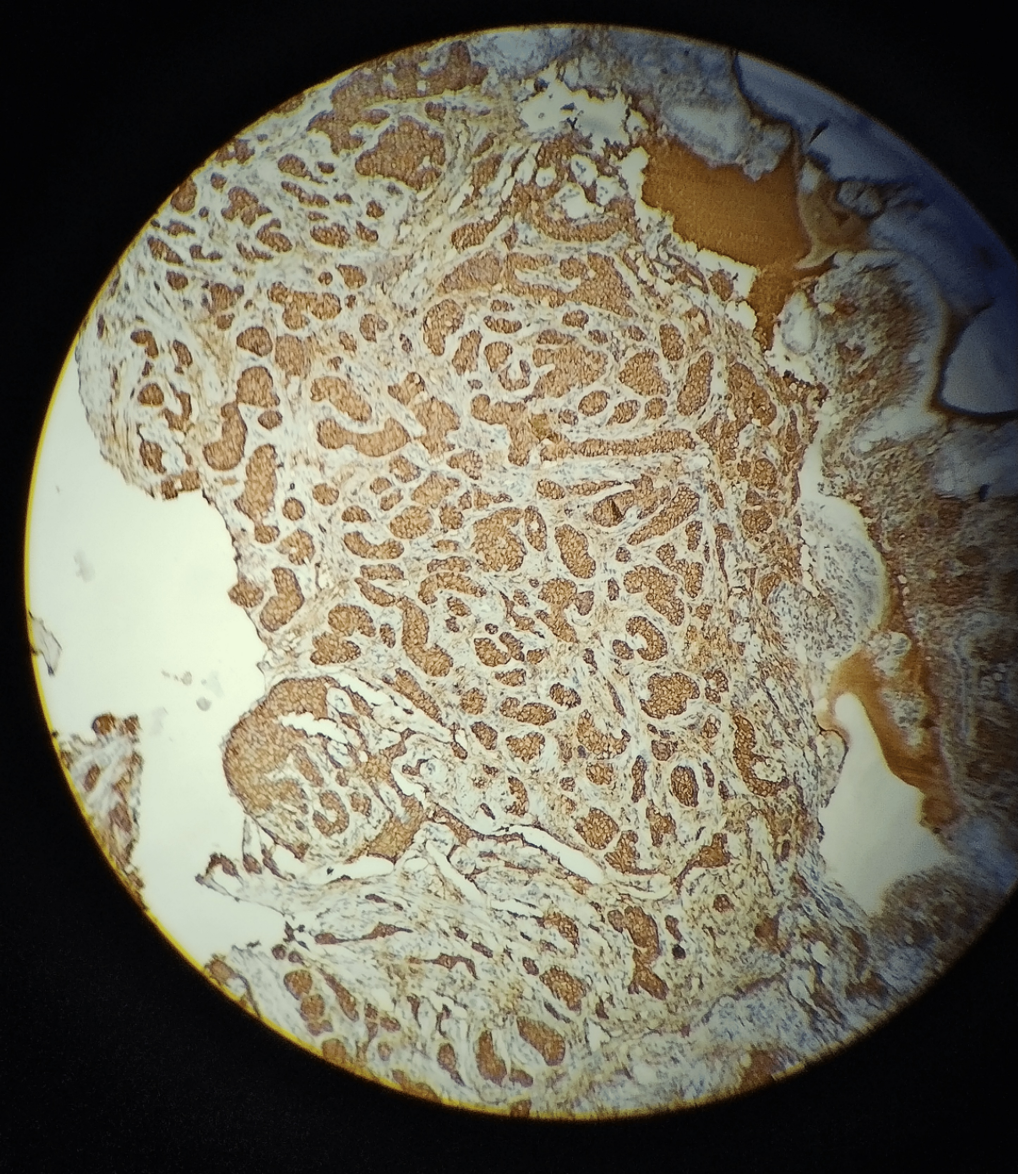
Microscopic view of a tissue sample stained with immunohistochemical markers. The brown coloration indicates positive staining, suggesting the presence of a specific protein, likely neuroendocrine cells, such as chromogranin A or synaptophysin.

Histological examination revealed neuroendocrine tumor cells, confirmed by positive staining for chromogranin A and synaptophysin and high Ki67, solidifying the diagnosis of a well-differentiated neuroendocrine tumor (Figures 3, 4).

**Figure 5:**
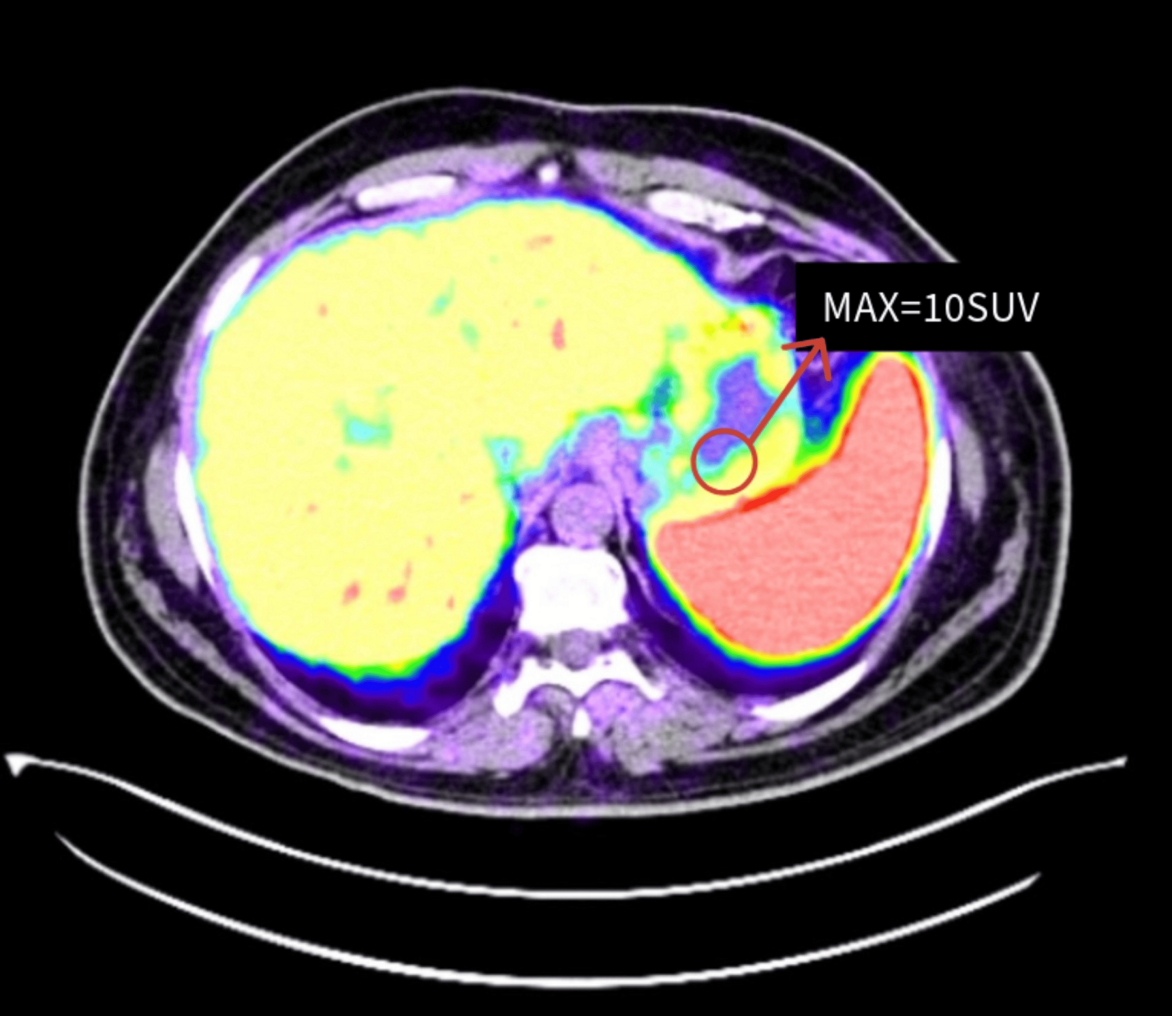
PET-DOTATATE scan revealing a metabolically active lesion with enhanced uptake indicating the presence of somatostatin receptors, suggestive of neuroendocrine tumor involvement. The PET-DOTATATE scan highlights a hypermetabolic nodule in the stomach with a maximum standardized uptake value (SUV) of 10, indicating increased somatostatin receptor activity, consistent with a gastric neuroendocrine tumor. The nodule is located near the greater curvature of the stomach and shows significant radiotracer uptake, confirming the presence of the neuroendocrine tumor.

As demonstrated above, endoscopic evaluation and biopsy confirmed a neuroendocrine tumor with a PET-DOTATATE gallium scan further confirming the active somatostatin receptor involvement showing an isolated nodule in greater curvature of the stomach (Figure 5).

Treatment

The patient was started on vitamin B12 injections due to the significant deficiency. Given the incidental discovery of the neuroendocrine tumor and the patient’s comorbid hepatitis B infection (viral load: 22,726 copies/mL), a multidisciplinary team involving surgery and gastroenterology was convened to determine the optimal management strategy. The decision was made to proceed with surgical intervention for the neuroendocrine tumor, considering the absence of metastatic disease and the tumor’s localized nature. The patient was also started on entecavir 0.5 mg to treat hepatitis B infection.

Surgery

The patient underwent resection of the tumor in the greater curvature of the stomach. The surgical margins were clear and the postoperative course was uneventful. He also continued with antiviral therapy for hepatitis B, given the high viral load to prevent potential liver-related complications.

Follow-up

The patient was placed under close surveillance with regular follow-up, Repeat serum vitamin B12 in the next follow-up was 489 pg/mL (normal range: 200-900 pg/mL), hemoglobin was 13.3 g/dL(normal range for men: 13.8-17.2 g/dL), MCV was 68.6 fL (normal range 60-100 fL), and liver function tests were within the normal limits.

Three months post-surgery, the patient remained asymptomatic with no signs of tumor recurrence. His anemia improved significantly with ongoing vitamin B12 supplementation, and liver function tests normalized. He was advised to continue antiviral therapy and was scheduled for follow-up investigations in six months.

## Discussion

This case illustrates the complex interplay between a rare GNET and severe vitamin B12 deficiency in a 48-year-old male from South India. The patient’s vegetarian diet likely contributed to the deficiency. The presentation included macrocytic anemia, mild hepatomegaly, pallor, and icterus with elevated liver enzymes likely due to concurrent hepatitis B infection [9].

The discovery of the GNET during an upper gastrointestinal endoscopy was incidental, underscoring that many of these tumors are asymptomatic. Biopsy, PET-DOTATATE gallium scan, and immunohistochemistry confirmed a localized tumor necessitating a multidisciplinary approach due to the patient’s high hepatitis B viral load [10].

The treatment strategy included surgical resection of the GNET, which was successful with clear margins and no metastasis, along with vitamin B12 supplementation. The postoperative course was uneventful, and antiviral therapy was initiated to manage the hepatitis B infection [11].

At the three-month follow-up, liver function tests and vitamin B12 levels returned to normal with no signs of cancer recurrence. This case highlights the importance of a thorough diagnostic evaluation in patients with vitamin B12 deficiency particularly vegetarians to effectively manage complex comorbidities. As demonstrated in this patient, a multidisciplinary approach is crucial for achieving optimal outcomes.

## Conclusions

This case highlights the importance of thorough examinations during standard medical examinations as unintentional discoveries can point to serious illnesses. A successful prognosis for this patient was largely dependent on early discovery and management of the neuroendocrine tumor in addition to a multidisciplinary approach. The administration of vitamin B12, tumor removal, and antiviral medication were directly associated with the patient’s clinical improvements, which included the normalization of anemia and liver function. The patient’s comorbidities were successfully treated by these therapeutic approaches, which aided in his general recovery.

A more thorough grasp of the ramifications of this case would be provided by investigating the long-term prognosis based on the available evidence. In complex situations combining various and seemingly unrelated illnesses, optimal outcomes require vigilance and comprehensive care.
